# Stereopsis impairment and its association with fovea-disc angle in congenital superior oblique palsy patients with compensatory head posture: a cross-sectional study

**DOI:** 10.3389/fmed.2026.1851621

**Published:** 2026-06-12

**Authors:** Yufei Wei, Song Mao, Wenli Lu, Meng Pan, Ailin Chen, Fang Gao, Shengyuan Chen, Xin Meng, Xuefeng Shi

**Affiliations:** 1 Clinical College of Ophthalmology, Tianjin Medical University, Tianjin, China; 2 Tianjin Key Laboratory of Ophthalmology and Visual Science, Tianjin Eye Institute, Tianjin Eye Hospital, Tianjin, China; 3 School of Public Health, Tianjin Medical University, Tianjin, China; 4 School of Medicine, Nankai University, Tianjin, China

**Keywords:** compensatory head posture, congenital superior oblique palsy, fovea-disc angle, imaging parameter, ocular torsion, stereopsis

## Abstract

**Purpose:**

To characterize stereopsis impairment in patients with congenital superior oblique palsy (CSOP) with compensatory head posture (CHP) and to explore the fovea-disc angle (FDA) as a potential imaging parameter for assessing binocular visual dysfunction.

**Methods:**

This cross-sectional study enrolled 20 CSOP patients with CHP and 20 age- and sex-matched controls between November 2024 and March 2026. Stereopsis was assessed using the Titmus Stereotest, Randot Stereotest (near), and Distance Randot Stereotest under both the CHP and the primary position. Stereopsis was compared between CSOP patients and controls, and between the CHP and the primary position within the CSOP group. Stereopsis was categorized as normal (≤100 arcsec) or impaired (>100 arcsec), and the proportions of CSOP patients with impaired distance and near stereopsis under the CHP were compared. FDA was measured using fundus photography and an imaging analysis software in patients with CSOP. Correlation analyses were performed to evaluate the associations between log-transformed stereopsis and clinical parameters.

**Results:**

CSOP patients with CHP showed significantly worse stereopsis than controls (Titmus: *U* = 20.00, *p* < 0.0001; Randot near: *U* = 63.00, *p* < 0.0001; Randot distance: *U* = 23.50, *p* < 0.0001). Within the CSOP group, near stereopsis was significantly better under the CHP than under the primary position (Titmus: *W* = 61.00, *p* = 0.004; Randot near: *W* = 80.00, *p* = 0.003), whereas distance stereopsis showed no significant improvement with CHP (*W* = 11.00, *p* = 0.250). Furthermore, the proportion of impaired distance stereopsis under the CHP remained high (85.00%, 17/20), whereas near stereopsis was impaired in only 35.00% (7/20) (*p* = 0.003). The binocular FDA was negatively correlated with both near (*r* = −0.50, 95% *CI*: −0.77 to −0.07, uncorrected *p* = 0.025) and distance (*r* = −0.48, 95% *CI*: −0.77 to −0.04, uncorrected *p* = 0.031) stereopsis under the CHP. No correlations were found between stereopsis and astigmatism, interocular differences in SE and astigmatism, or inferior oblique overaction (all *p* > 0.05).

**Conclusion:**

CSOP patients with CHP exhibit impairment in both distance and near stereopsis, with distance stereopsis being more severely affected. Binocular FDA is negatively correlated with stereopsis and may serve as a promising imaging parameter for assessing binocular visual dysfunction in CSOP with CHP.

## 1 Introduction

Stereopsis is the highest form of binocular visual function, enabling the perception of depth through the neural integration of disparate retinal images from both eyes. This capacity is fundamental for fine motor coordination, precise hand-eye coordination, and spatial navigation in daily activities (1). There is evidence demonstrating that impaired stereopsis compromises gross motor skills and daily living tasks across all age groups (2).

Congenital superior oblique palsy (CSOP) is a common type of paralytic and vertical strabismus characterized by superior oblique paresis, resulting in vertical deviation and excyclotorsion of the affected eye (3, 4). To maintain binocular single vision, patients frequently adopt a compensatory head posture (CHP), typically characterized by head tilt, chin depression, and face turn, thereby minimizing vertical deviation and excyclotorsion (5, 6). Previous research on CSOP has primarily focused on surgical outcomes in terms of ocular alignment and correction of head posture (7, 8). However, the impact of CSOP on binocular visual function remains incompletely characterized. Specifically, the extent to which the CHP preserves stereopsis has not been well established.

The fovea-disc angle (FDA) is defined as the angle between the horizontal line passing through the optic disc center and the line connecting the disc center to the fovea. This anatomical relationship can be readily assessed using fundus photography combined with imaging analysis software, providing an objective, noninvasive, and reproducible method for quantifying ocular torsion. Previous studies have primarily focused on the application of FDA to analyze ocular torsion, evaluate surgical outcomes, or adjust optical coherence tomography (OCT) scans in glaucoma (9–11). However, the relationship between FDA and stereopsis impairment in patients with CSOP remains insufficiently investigated.

This cross-sectional study aimed to address these gaps by investigating stereopsis impairment in CSOP patients with CHP and evaluating the FDA as a potential objective imaging parameter for binocular visual dysfunction in these patients. These findings may provide evidence to support the routine stereopsis assessment and FDA measurement as complementary tools for clinical assessment and intervention in this population.

## 2 Materials and methods

### 2.1 Study design and participants

**Figure 1:**
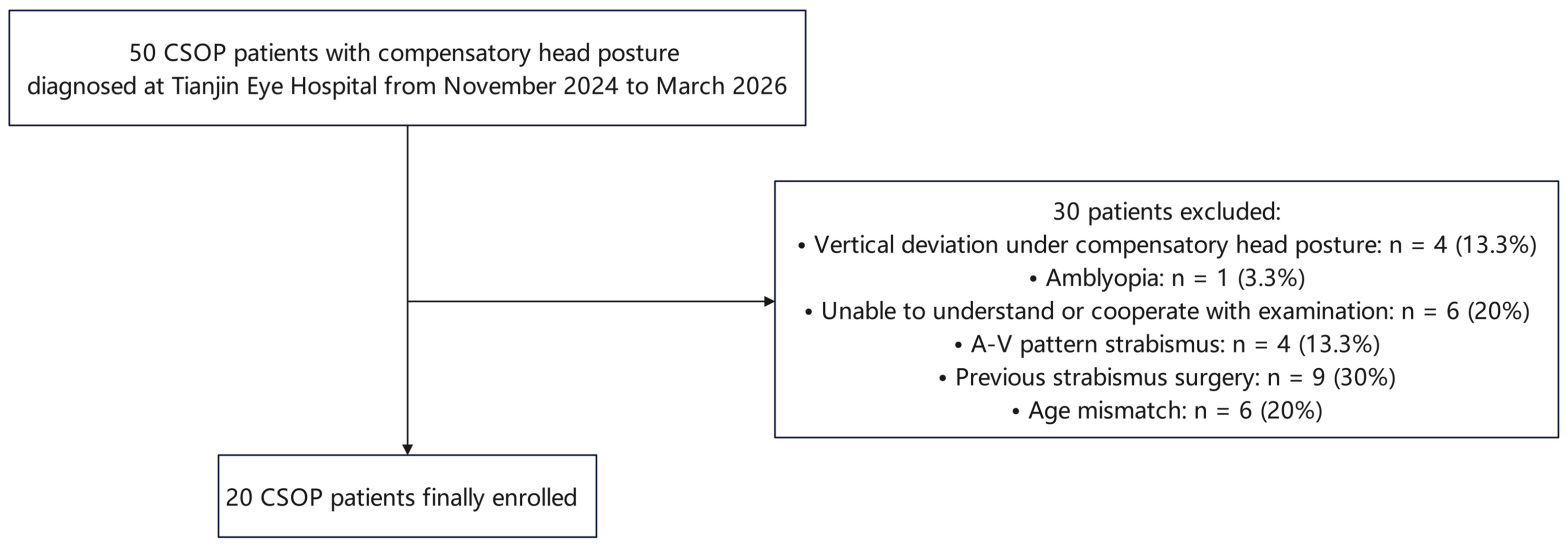
Flowchart of patient enrollment and exclusion. CSOP, Congenital superior oblique palsy.

This was a cross-sectional study. A consecutive sample of 50 CSOP patients with CHP was recruited from the Tianjin Eye Hospital between November 2024 and March 2026. Twenty patients with CSOP met all the criteria and were ultimately enrolled in the study (Figure 1).

The inclusion criteria were as follows: (1) meeting the diagnostic criteria for congenital superior oblique palsy, with the presence of CHP and no vertical deviation under this posture; (2) best-corrected visual acuity within the normal range for their age (12, 13); (3) spherical equivalent (SE) within ±5.00 D in both eyes, with no significant interocular difference in SE (less than 1.00 D) and no other risk factors for amblyopia; and (4) ability to understand and cooperate with stereopsis and refractive examinations.

The exclusion criteria were as follows: (1) acquired superior oblique palsy due to trauma, ischemia, or other factors; (2) combined with horizontal strabismus, restricted ocular motility, concomitant strabismus, or presence of nystagmus; (3) coexisting ocular organic diseases or systemic conditions that might affect binocular visual function; (4) history of prior strabismus surgery; and (5) inability to understand or cooperate with stereopsis and refractive examinations.

During the same period, 20 healthy controls were recruited from the same institution and matched by age (± 2 years) and sex in a 1:1 ratio (14, 15). The control participants had no history of strabismus or other ocular diseases and met the refractive and visual acuity criteria applied to the CSOP group. They also had no prior history of strabismus surgery or amblyopia treatment and no organic ocular diseases or systemic conditions that might affect binocular vision function.

The study was conducted in accordance with the Declaration of Helsinki and approved by the Ethics Committee of Tianjin Eye Hospital (approval number: KY-2025033). All participants were informed of the study contents, and written informed consent was obtained from the parents or legal guardians of the pediatric patients. This study was designed and reported in accordance with the Strengthening the Reporting of Observational Studies in Epidemiology (STROBE) guidelines.

### 2.2 Data collection and ophthalmic examinations

All examinations were performed by experienced ophthalmologists and optometrists. All participants underwent comprehensive demographic data collection (including age and sex), as well as external eye, anterior segment, and fundus examinations to rule out organic eye diseases. For patients with CSOP, additional clinical information was collected, including presenting symptoms, age of onset, disease duration, and characteristics of CHP.

Cycloplegic refraction was performed in patients aged ≤14 years. Refractive error was converted to SE using the formula: SE = sphere + 1/2 cylinder. Interocular differences were calculated as right minus left differences.

All examinations were performed by a single, experienced ophthalmologist. Deviation angles were measured using a prism and alternate cover test at both distance (5 m) and near (33 cm), with fixation by the non-paralytic eye and the paralytic eye, respectively. Extraocular muscle function was assessed through ocular motility examination and the Bielschowsky head tilt test. The degree of inferior oblique overaction was graded on a scale of +1 to +4 (16). The characteristics of the CHP were observed and documented.

All stereopsis examinations were performed by a single, experienced ophthalmologist. Following full refractive correction, stereopsis was evaluated with participants wearing polarized glasses that were adjusted to ensure proper fit over both eyes. Near stereopsis was assessed using the Titmus Stereo Test (Stereo Optical Co., Inc., USA) and the Randot Stereotest (Stereo Optical Co., Inc., USA) at a viewing distance of 40 cm. To minimize false-positive responses, the examiner inverted the stereotest booklet or rotated it by 90° and inquired whether the patient perceived a recessed circle or no depth. Distance stereopsis was assessed using the Distance Randot Stereotest (Vision Assessment Corporation, USA) at 3 m distance. The Titmus Stereo Test, the Randot Stereotest, and the Distance Randot Stereotest were performed sequentially. Stereopsis was measured in CSOP patients under both the CHP and the primary position, and in controls under the primary position. The stereotests were performed first under the primary position and then under the CHP. A 1-min rest interval was provided between the test positions and between the different stereotests. For testing under the CHP, the stereotest booklet was oriented perpendicular to the participant’s line of sight while preserving the alignment of the polarization axes. CHP was maintained throughout the testing period using verbal reminders and gentle physical guidance, if necessary. The finest disparity correctly identified was recorded as the stereoacuity threshold. Based on the results, stereopsis was categorized as normal (stereoacuity ≤100 arcsec) or impaired (stereoacuity >100 arcsec) (17–19).

**Figure 2:**
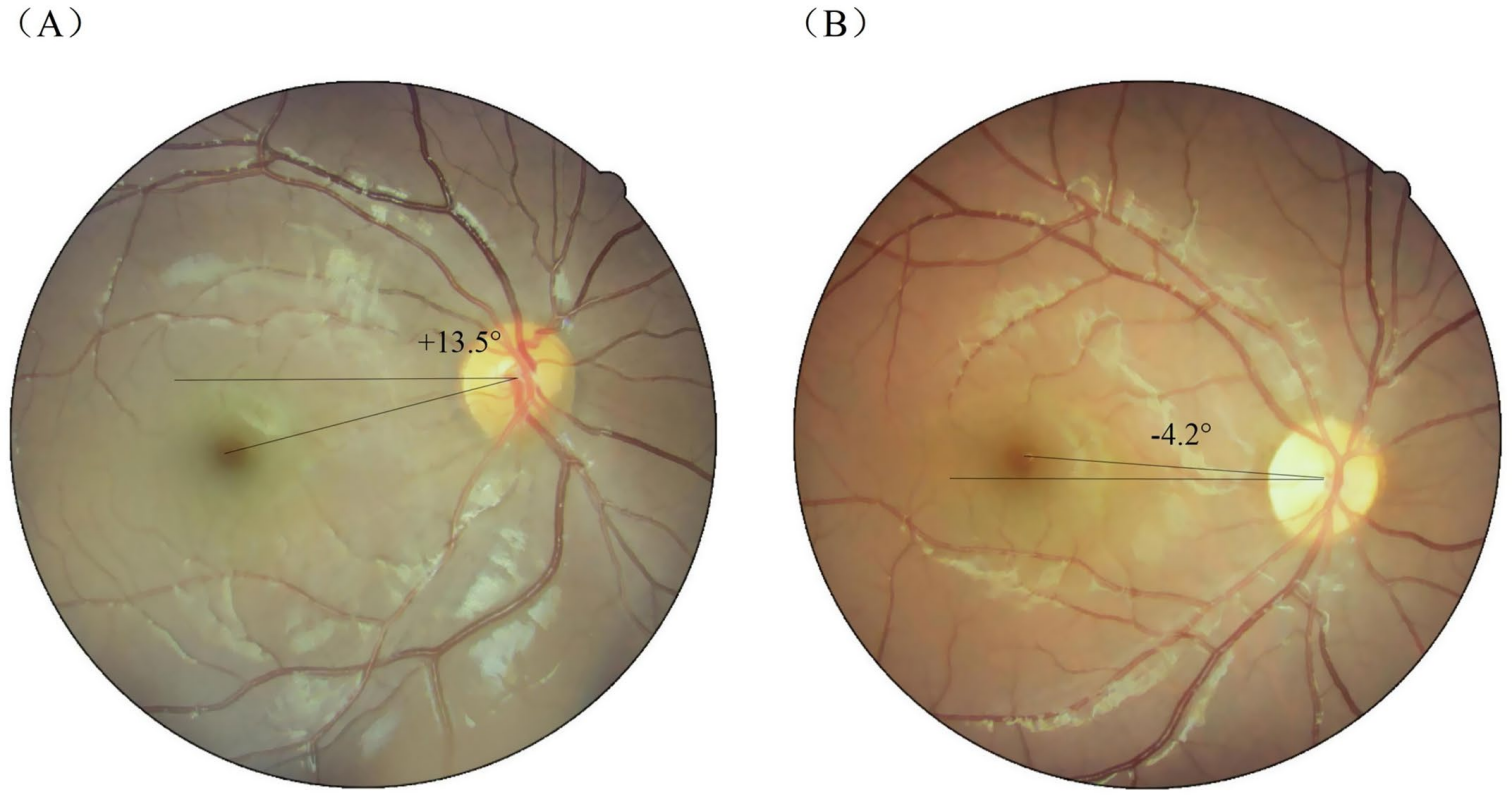
Schematic diagram of the measurement method for FDA. **(A)** Fundus excyclotorsion: the fovea is located below the horizontal line through the center of the optic disc, marked as “+”; **(B)** Fundus incyclotorsion: the fovea is located above the horizontal line through the center of the optic disc, marked as “−.” FDA, fovea-disc angle.

Fundus photographs were obtained using a non-mydriatic fundus camera (CR-2 AF, Canon, Japan). With the chin positioned on the chin rest and the forehead against the forehead strap, the patients fixated on an internal target while bilateral fundus images were captured in the primary position. The images were imported into Adobe Photoshop CC 2018 (Adobe Systems Inc., USA). A horizontal reference line was manually drawn through the center of the optic disc, and a second line connecting the center of the optic disc to the fovea was created. The software automatically calculated the angle between these two lines, which was defined as the fovea-disc angle (FDA). Excyclotorsion was denoted as positive (+) when the fovea was located below the horizontal reference line, and incyclotorsion as negative (−) when the fovea was located above it (Figure 2). FDA measurements were performed by a single experienced ophthalmologist. Each eye was measured three times, and the average value was used to represent the objective torsion of the eye. The sum of the objective torsion in both eyes was also recorded.

### 2.3 Statistical analysis

Statistical analyses were performed using GraphPad Prism version 10.1 (GraphPad Software, San Diego, CA, USA). Continuous variables were first tested for normality using the Shapiro–Wilk test. Normally distributed variables are presented as mean ± standard deviation (SD). Comparisons between two groups were conducted using the independent-samples *t* test, and within group comparisons were performed using the paired *t* test. Non-normally distributed variables are expressed as medians (interquartile range, IQR). Between group comparisons were analyzed using the Mann–Whitney *U* test, and within group comparisons were assessed using the Wilcoxon signed-rank test. Categorical variables are presented as frequencies and percentages (*n*, %) and were compared using Fisher’s exact test. A *p* value < 0.05 was considered statistically significant.

To assess the intra-observer repeatability of the FDA measurements, the intraclass correlation coefficient (ICC) with a two-way random-effects model for absolute agreement was calculated. ICC > 0.7 was considered significant, indicating good agreement between the three repeated FDA measurements. The intra-observer average-measures ICC was 0.998 [95% confidence interval (*CI*): 0.996–0.999] for the paretic eye and 0.999 (95% *CI*: 0.998–1.000) for the non-paretic eye, indicating excellent repeatability.

All stereoacuity values were log10 transformed. For patients with undetectable stereopsis, a value corresponding to the next logarithmic step above the maximum disparity tested was assigned based on the grading scale (20).

Exploratory Pearson or Spearman correlation analyses were used to assess the relationships between log-transformed stereoacuity and variables including age, refractive error, vertical deviation in the primary position and FDA. Uncorrected *p* values were reported.

## 3 Results

### 3.1 Comparison of general data between the CSOP group and the control group

Among the 20 CSOP patients with CHP, there were 13 males and 7 females, with an age range of 3 to 14 years and a mean age of 7.10 ± 3.06 years. The control group also consisted of 20 individuals (13 males and 7 females), with an age range of 3 to 14 years and a mean age of 7.10 ± 2.81 years. No statistically significant differences were observed between the two groups in terms of age or sex (age: *t* = 0.00, *p* > 0.999; sex: *p* > 0.999).

The mean SE was 0.71 ± 2.05 diopters (D) in the right eye and 0.57 ± 2.10 D in the left eye for the CSOP group, and 0.23 ± 1.62 D and 0.21 ± 1.44 D, respectively, for the control group. In the CSOP group, there was no statistically significant difference in the SE between the two eyes (*W* = −19.00, *p* = 0.426). Similarly, no statistically significant difference was found in the control group (*W* = 14.00, *p* = 0.697). Comparisons between the two groups for the corresponding eyes also revealed no statistically significant differences (right eye: *t* = 0.82, *p* = 0.416; left eye: *t* = 0.64, *p* = 0.528).

The median (IQR) astigmatism was 0 (−0.19, 0.69) D in the right eye and 0 (−0.19, 0.75) D in the left eye for the CSOP group, and 0.25 (0, 1.19) D and 0.25 (0, 0.69) D, respectively, for the control group. Regarding astigmatism, no statistically significant difference was observed between the two eyes in the CSOP group (*W* = −5.00, *p* = 0.781), nor in the control group (*W* = −13.00, *p* = 0.488). Comparisons between the two groups for the corresponding eyes also showed no statistically significant differences (right eye: *U* = 158.00, *p* = 0.249; left eye: *U* = 172.00, *p* = 0.445) (Supplementary Table S1).

### 3.2 Vertical deviation in CSOP patients with CHP

At distance, the vertical deviation in the primary position was 11.85 ± 8.63 prism diopters (PD) when fixating with the paretic eye and 13.90 ± 8.96 PD when fixating with the non-paretic eye, with no statistically significant difference between the two fixation conditions (*W* = 24.00, *p* = 0.172). At near, the vertical deviation in the primary position was 11.15 ± 8.71 PD when fixating with the paretic eye and 13.40 ± 8.82 PD when fixating with the non-paretic eye, also showing no statistically significant difference (*W* = 42.00, *p* = 0.062). No statistically significant differences were observed when comparing the vertical deviation in the primary position for the same fixating eye at different testing distances (paretic eye fixation: *W* = −18.00, *p* = 0.172; non-paretic eye fixation: *W* = −14.00, *p* = 0.313). All participants had zero vertical deviation under the CHP for both distance and near fixation.

### 3.3 Comparison of stereopsis between the CSOP group and the control group

**Figure 3:**
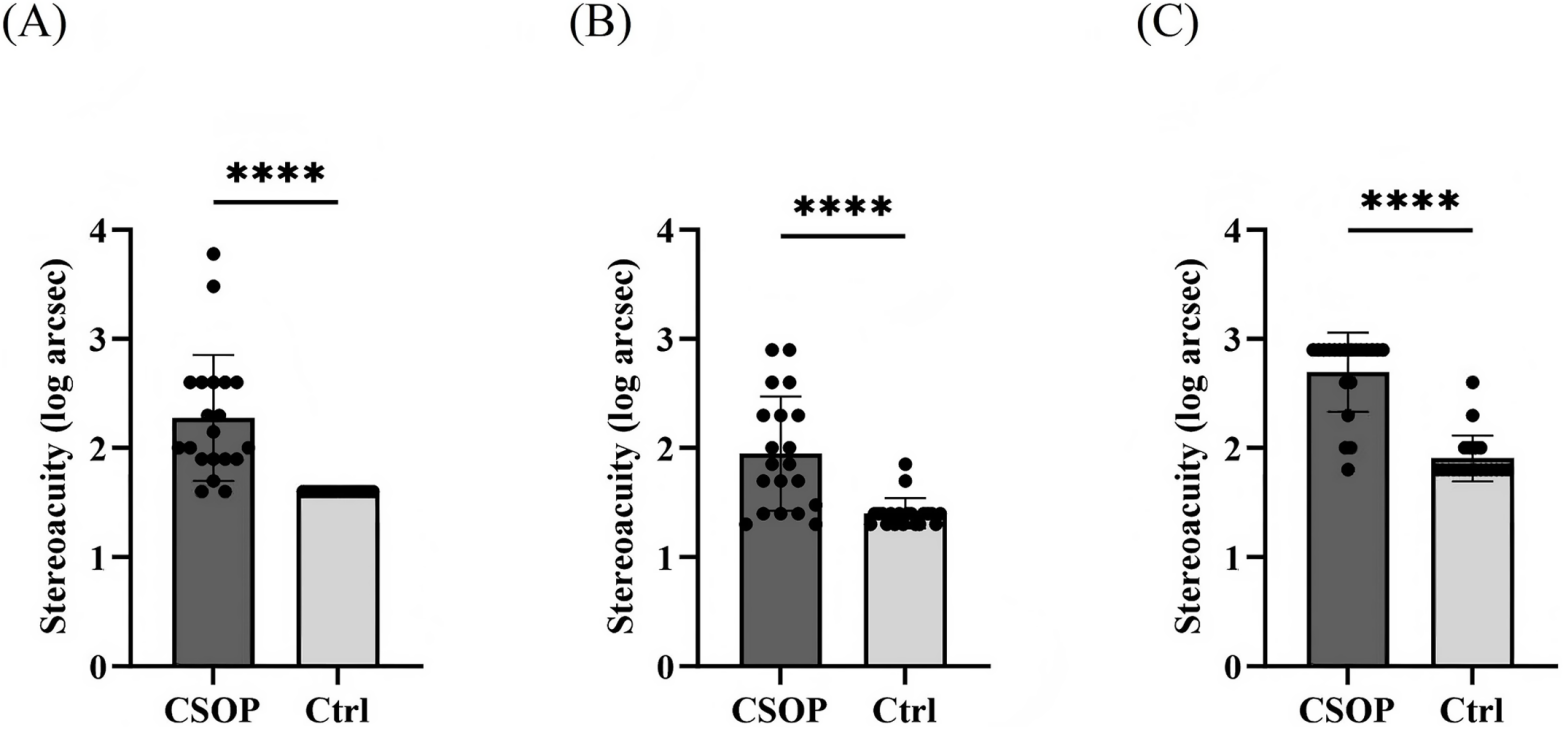
Comparison of stereopsis between the group of CSOP with CHP and the normal control group. **(A)** Comparison of Titmus stereopsis between the two groups; **(B)** Comparison of Randot near stereopsis between the two groups; **(C)** Comparison of Randot distance stereopsis between the two groups. CSOP, congenital superior oblique palsy; CHP, compensatory head posture. Stereopsis in the CSOP group was assessed under compensatory head posture. ****p < 0.0001.

However, the CSOP patients demonstrated significantly worse stereopsis across all measurements under the CHP compared to the control group (Titmus: *U* = 20.00, *p* < 0.0001; Randot near stereotest: *U* = 63.00, *p* < 0.0001; Randot distance stereotest: *U* = 23.50, *p* < 0.0001). In the CSOP group, the median (IQR) Titmus stereopsis was 2.08 (1.90, 2.60) log arcsec, Randot near stereopsis was 1.85 (1.42, 2.30) log arcsec, and Randot distance stereopsis was 2.90 (2.60, 2.90) log arcsec under the compensatory head posture. In the control group, corresponding values were 1.60 (1.60, 1.60) log arcsec, 1.40 (1.30, 1.40) log arcsec, and 1.80 (1.80, 2.00) log arcsec, respectively (Supplementary Table S1; Figure 3).

### 3.4 Comparison of stereopsis under the CHP versus the primary position in CSOP patients

Within the CSOP group, near stereopsis was significantly better when measured under the CHP than under the primary position (Titmus: *W* = 61.00, *p* = 0.004; Randot near stereotest: *W* = 80.00, *p* = 0.003). In contrast, no significant difference was observed in distance stereopsis between the two conditions (*W* = 11.00, *p* = 0.250).

Notably, the proportion of CSOP patients with impaired distance stereopsis remained high under both two conditions, affecting 85.00% (17/20) of patients under the CHP and 90.00% (18/20) under the primary position. The proportion of patients with impaired near stereopsis under the CHP was 35.00% (7/20), which was significantly lower than the proportion with impaired distance stereopsis under the same condition (*p* = 0.003) (Supplementary Table S2).

### 3.5 Correlation analysis of stereopsis with clinical factors in CSOP patients with CHP

**Figure 4:**
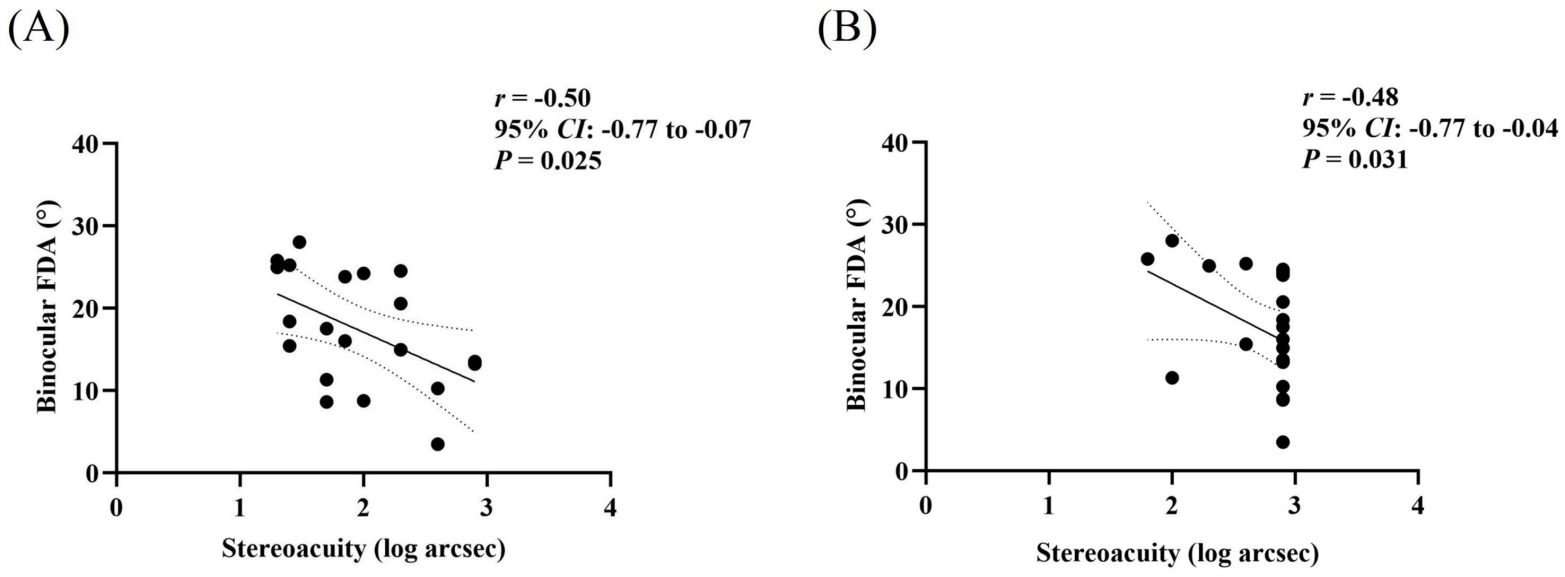
Correlation between binocular FDA and stereopsis in CSOP patients with CHP. **(A)** Correlation between binocular FDA and Randot near stereopsis; **(B)** Correlation between binocular FDA and Randot distance stereopsis. Higher log stereoacuity values indicate worse stereopsis; therefore, the observed negative correlation indicates that a smaller binocular FDA is associated with relatively worse stereopsis in our cohort. FDA, fovea-disc angle; CSOP, congenital superior oblique palsy; CHP, compensatory head posture. Uncorrected *p* values are shown for exploratory purposes.

**Figure 5:**
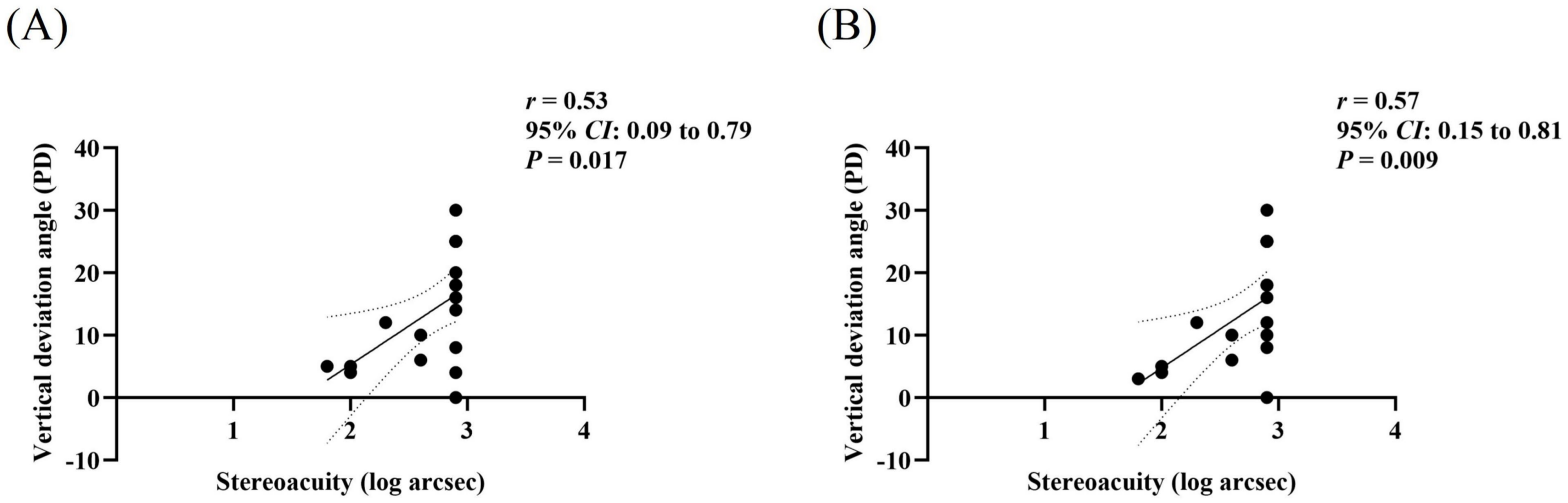
Correlation between vertical deviation angle in the primary position (non-paralytic eye fixation) and stereopsis in CSOP patients with CHP. **(A)** Correlation between distance vertical deviation angle in the primary position (non-paralytic eye fixation) and Randot distance stereopsis; **(B)** Correlation between near vertical deviation angle in the primary position (non-paralytic eye fixation) and Randot distance stereopsis. PD, prism diopters; CSOP, congenital superior oblique palsy; CHP, compensatory head posture. Uncorrected *p* values are shown for exploratory purposes.

In CSOP patients with CHP, the FDA was 9.03 ± 4.66° for the paretic eye and 8.39 ± 5.93° for the non-paretic eye, with no statistically significant difference between the two eyes (*t* = 0.36, *p* = 0.725). The total FDA for both eyes was 17.41 ± 6.99° (Supplementary Table S3). Notably, the total binocular FDA was negatively correlated with log-transformed near and distance stereopsis under the CHP (Randot near stereotest: *r* = −0.50, 95% *CI*: −0.77 to −0.07, uncorrected *p* = 0.025; Randot distance stereotest: *r* = −0.48, 95% *CI*: −0.77 to −0.04, uncorrected *p* = 0.031) (Figure 4). Log-transformed near stereopsis under the CHP also showed negative correlations with age and FDA of the paralytic eye, and a positive correlation with SE of the right eye (all uncorrected *p* < 0.05). Vertical deviation angles in the primary position with fixation by the non-paralytic eye were positively correlated with log-transformed distance stereopsis under the CHP (all uncorrected *p* < 0.05) (Figure 5). No correlations were found between stereopsis and astigmatism, interocular differences in SE and astigmatism, or inferior oblique overaction (all *p* > 0.05) (Supplementary Table S4).

## 4 Discussion

Although previous studies have investigated preoperative binocular visual function in patients with CSOP, none have performed stratified analyses according to the presence or absence of CHP to evaluate binocular visual function impairment in these patients. Moreover, the characteristics of binocular visual function and its influencing factors in CSOP patients with CHP have not been fully elucidated yet. In the present study, distance and near stereopsis were systematically assessed in CSOP patients with CHP under different head position conditions. The results indicated that, compared with age- and sex-matched normal controls, CSOP patients exhibited varying degrees of impairment in both distance and near stereopsis while maintaining CHP, with distance stereopsis being more severely affected. In addition, this study found that the binocular FDA was negatively correlated with stereopsis, suggesting that a smaller binocular FDA was associated with relatively worse stereopsis in CSOP patients with CHP. This finding suggests that FDA may serve as a promising imaging parameter for assessing binocular visual function impairment in CSOP patients.

CHP is an important mechanism that allows CSOP patients to maintain binocular single vision. Through head tilt, chin depression, and facial turn, the CHP reduces vertical deviation and ocular extorsion, thereby creating favorable conditions for binocular fusion (6, 21). Although previous studies have quantified the relationship between the degree of head tilt and vertical deviation, the role of the CHP in preserving stereopsis remains underexplored (22). One study reported the presence rate of stereopsis in CSOP patients under the CHP; however, it relied solely on contour stereopsis tests and did not characterize the extent of stereopsis impairment (23). In the present study, we found that near stereopsis in CSOP patients under the CHP was significantly better than in the primary position, suggesting that CHP exerts a protective effect on near stereopsis. In contrast, distance stereopsis was severely impaired in both head positions, with no significant difference between CHP and the primary position. However, this non-significant result should be interpreted with caution because of the potential floor effect of the distance stereopsis test (24). The Distance Randot Stereotest used in this study has a measurement range of 400 to 63 arcsec, with values worse than 400 arcsec recorded as nil stereoacuity. A substantial proportion of our CSOP patients exhibited distance stereopsis at or below this minimum threshold, leading to clustering at the measurement floor. This floor effect limits the dynamic range of the test for detecting subtle differences, and a true but modest improvement in distance stereopsis with CHP could remain undetected.

In this study, by comparing the proportions of patients with impaired Randot distance stereopsis and Randot near stereopsis among CSOP patients under the CHP, we found that the proportion of patients with impaired distance stereopsis was significantly higher than that with impaired near stereopsis. These findings indicate that CSOP patients with CHP exhibit impairment in both distance and near stereopsis, with distance stereopsis being more severely affected. This phenomenon may be explained by the mechanism of accommodative convergence linkage (25, 26). During near-fixation, the synergistic interaction between accommodation and convergence is enhanced, facilitating better ocular alignment control and maintenance of binocular fusion, thereby partially mitigating stereopsis impairment caused by vertical deviation and ocular extorsion. In contrast, during distance fixation, the accommodative demand decreases, the compensatory effect of accommodative convergence weakens, and ocular alignment control declines, making binocular visual function more vulnerable to vertical deviation and extorsion. Consequently, stereopsis is more severely impaired. These results suggest that greater attention should be paid to distance stereopsis in clinical evaluation, as its impairment may be more subtle and less amenable to compensation by the CHP.

In patients with CSOP, superior oblique muscle insufficiency may lead to ocular extorsion (27, 28). As an objective metric for quantifying ocular torsion, the FDA has been used primarily to assess ocular torsion and monitor surgical outcomes (29–32). A previous study in patients with intermittent exotropia found that FDA was positively correlated with stereopsis, indicating that greater ocular torsion was associated with poorer stereopsis (33). However, in the present study, we observed a significant negative correlation between binocular FDA and both near and distance stereopsis in patients with CSOP; specifically, a larger binocular FDA was associated with relatively preserved stereopsis. Interestingly, a larger binocular FDA was associated with relatively better stereopsis rather than worse stereopsis. To interpret this seemingly paradoxical finding, we propose the following hypothesis. When the paretic eye exhibits mild superior oblique paresis with some residual function, according to Hering’s law of equal innervation, the yoke muscle (contralateral inferior rectus) may become secondarily overactive, leading to increased excyclotorsion of the fellow eye. This results in a larger excyclotorsion of the fellow eye’s FDA and, consequently, a larger binocular FDA. At this stage, vertical deviation angles remain relatively small, and stereopsis impairment remains relatively mild. As the paresis progresses further, the ipsilateral inferior rectus (the synergist of the paretic superior oblique) may develop compensatory overaction when fixating with the paretic eye, and the fellow eye’s superior oblique may become secondarily overactive as well. This may increase the incyclotorsion of the fellow eye, partially or completely counteracting its excyclotorsion, thereby reducing the fellow eye’s FDA excyclotorsion and the binocular FDA. At this advanced stage, because vertical deviation increases and binocular alignment is more severely disrupted, stereopsis impairment becomes more pronounced, despite the reduction in FDA (Supplementary Figure S1). Notably, this reduction was relative, as CSOP patients still exhibited net excyclotropia compared with normal controls (Supplementary Figure S2). Taken together, the negative correlation between binocular FDA and stereopsis impairment in patients with CSOP may reflect the complex modulation of ocular torsion by secondary extraocular muscle changes at different stages of superior oblique paresis. These findings suggest that the FDA not only reflects anatomical changes in ocular torsion but may also serve as a promising objective imaging parameter for predicting binocular visual function. In particular, in strabismic conditions accompanied by ocular extorsion, such as CSOP, the FDA has considerable potential as an objective adjunctive tool for functional assessment, especially in non-cooperative child patients, offering the advantages of easy performance and good reproducibility based on fundus photography combined with image analysis software.

This study has several limitations. First, the cross-sectional design precludes causal inference and limits the assessment of the long-term effects of CHP and FDA on stereopsis. However, it provides a necessary foundation for generating hypotheses and designing longitudinal studies. Second, to ensure data quality and minimize confounding factors, some patients with CSOP who were too young to cooperate with stereopsis and refractive examinations or who had co-existing anisometropia or horizontal strabismus were excluded. While this inevitably reduced the sample size, it also enhanced the internal validity of the findings. Third, the clinical manifestations of patients with CSOP are diverse and, according to the Knapp classification, can be categorized into seven types. Some patients may also present with other co-existing strabismus. However, our findings were derived from a specific subset of CSOP patients with CHP and may not be generalizable to a broader CSOP population. Future multicenter, prospective studies with larger cohorts are warranted to validate the associations observed in this study and to explore the characteristics of stereopsis impairment and the predictive value of FDA across clinical subtypes.

## 5 Conclusion

This cross-sectional study indicates that CSOP patients with CHP exhibit significant impairment in both near and distance stereopsis, with distance stereopsis disproportionately affected. The identification of binocular FDA as a negative correlate of stereopsis suggests that a smaller binocular FDA is associated with relatively worse stereopsis in CSOP patients with CHP. These findings enhance our understanding of the sensory consequences of CSOP and suggest potential applications for FDA measurement in diagnosis and longitudinal monitoring. Further validation through prospective, longitudinal studies is warranted to explore these findings in larger cohorts and guide therapeutic interventions.

## Data Availability

The original contributions presented in the study are included in the article/Supplementary material, further inquiries can be directed to the corresponding author.

## References

[1] Al-Saud LM Mushtaq F Mirghani IA Balkhoyor A Keeling A Manogue M . Drilling into the functional significance of stereopsis: the impact of stereoscopic information on surgical performance. Ophthalmic Physiol Opt. (2017) 37:498–506. doi: 10.1111/opo.12393, 28656672 PMC5519940

[2] Smith D Ropar D Allen HA. Does stereopsis account for the link between motor and social skills in adults? Mol Autism. (2018) 9:55. doi: 10.1186/s13229-018-0234-4, 30386542 PMC6201514

[3] Dosunmu EO Hatt SR Leske DA Hodge DO Holmes JM. Incidence and etiology of presumed fourth cranial nerve palsy: a population-based study. Am J Ophthalmol. (2018) 185:110–4. doi: 10.1016/j.ajo.2017.10.019, 29102606 PMC5784757

[4] Farvardin H Ebrahimi F Talebnejad M Farvardin H Attar A Farvardin M. Three inferior oblique weakening procedures for management of mild hypertropia in unilateral superior oblique muscle palsy. J Ophthalmic Vis Res. (2024) 19:459–67. doi: 10.18502/jovr.v19i4.14394, 39917453 PMC11795008

[5] Kushner BJ. The influence of head tilt on ocular torsion in patients with superior oblique muscle palsy. J AAPOS. (2009) 13:132–5. doi: 10.1016/j.jaapos.2008.10.012, 19157937

[6] Akbari MR Khorrami-Nejad M Kangari H Akbarzadeh Baghban A Ranjbar PM. Ocular abnormal head posture: a literature review. J Curr Ophthalmol. (2022) 33:379–87. doi: 10.4103/joco.joco_114_20, 35128182 PMC8772496

[7] Huang L Wu Y Li N. A single inferior rectus muscle surgery for treatment of congenital superior oblique palsy with small deviation in primary position. Eur J Ophthalmol. (2021) 32:1174–7. doi: 10.1177/11206721211014377, 33938310

[8] Brodsky MC. Temporal slant recession of the inferior rectus muscle: a simple surgical treatment for diplopia caused by small vertical deviations. J Neuroophthalmol. (2023) 43:406–9. doi: 10.1097/WNO.0000000000001677, 35947106

[9] Miyata M Yoshikawa M Ohtsuki H Muraoka Y Hata M Yokota S . Age-related change and sex difference over 60s in disc-fovea angle in japanese population: the Nagahama study. Acta Ophthalmol. (2018) 96:e840–5. doi: 10.1111/aos.13642, 29369505

[10] Abri Aghdam K Katiraee A Chaibakhsh S Soltan Sanjari M Miraftabi A Nadjafi F . Evaluating ocular torsion following inferior oblique weakening in superior oblique palsy: a pilot study using color fundus photography and spectral domain optical coherence tomography. BMC Ophthalmol. (2025) 25:389. doi: 10.1186/s12886-025-04205-6, 40596931 PMC12220215

[11] Ordon AJ Simiera J Rosa A Loba P. Assessment of objective cyclotorsion changes following symmetric and asymmetric graded recession of inferior oblique muscle for v-pattern strabismus. Clin Ophthalmol. (2025) 19:3773–81. doi: 10.2147/OPTH.S543530, 41111596 PMC12533728

[12] Pang Y Lyons SA Chaplin PKN Block SS Fishman D Ciner EB. Recommended practices for vision screening in pre-school-age children: a 2025 update. Optom Vis Sci. (2025) 102:589–95. doi: 10.1097/OPX.0000000000002290 40892439 PMC12520030

[13] Cruz OA Repka MX Hercinovic A Cotter SA Lambert SR Hutchinson AK . Amblyopia preferred practice pattern. Ophthalmology. (2023) 130:P136–78. doi: 10.1016/j.ophtha.2022.11.003, 36526450 PMC10701408

[14] Grimes DA Schulz KF. Compared to what? Finding controls for case-control studies. Lancet. (2005) 365:1429–33. doi: 10.1016/S0140-6736(05)66379-9, 15836892

[15] Taylor JM. Choosing the number of controls in a matched case-control study, some sample size, power and efficiency considerations. Stat Med. (1986) 5:29–36. doi: 10.1002/sim.4780050106, 3961313

[16] Gim Y Kim SJ. The long-term surgical outcomes for the treatment of inferior oblique overaction: a retrospective single-center study. Sci Rep. (2025) 15:23797. doi: 10.1038/s41598-025-93497-1, 40610606 PMC12229658

[17] Xu L Liu L Wu H. Evaluation of the relationship between aniseikonia and stereopsis using a new method. Front Med (Lausanne). (2022) 9:889398. doi: 10.3389/fmed.2022.889398, 35669921 PMC9163365

[18] Xu Z Wu Z Wen Y Ding M Sun W Wang Y . Prevalence of anisometropia and associated factors in Shandong school-aged children. Front Public Health. (2022) 10:1072574. doi: 10.3389/fpubh.2022.1072574, 36620276 PMC9815018

[19] Elamurugan V Shankaralingappa P Aarthy G Kasturi N Babu RK. Assessment of stereopsis in pediatric and adolescent spectacle-corrected refractive error—a cross-sectional study. Indian J Ophthalmol. (2022) 70:604–8. doi: 10.4103/ijo.IJO_997_21, 35086245 PMC9024002

[20] Lee HJ Kim SJ Yu YS. Stereopsis in patients with refractive accommodative esotropia. J AAPOS. (2017) 21:190–5. doi: 10.1016/j.jaapos.2017.05.009, 28532705

[21] Khorrami-Nejad M Akbari MR Shakor YA Kangari H. Assessing facial asymmetry before and after early treatment of abnormal head posture caused by ocular problems. Clin Exp Optom. (2026):1–10. doi: 10.1080/08164622.2026.2631013, 41730263

[22] Akbari MR Khorrami-Nejad M Kangari H Baghban AA Raeesdana K Ranjbar-Pazooki M. The correlation between hypertropia and head tilt in congenital unilateral superior oblique muscle palsy. J Curr Ophthalmol. (2021) 33:336–41. doi: 10.4103/joco.joco_60_20, 34765824 PMC8579784

[23] Erduran B Niyaz ŞL. Screening of clinical data of patients with abnormal head posture and investigation of abnormal head posture change after treatment. Turk J Ophthalmol. (2025) 55:11–5. doi: 10.4274/tjo.galenos.2024.71163, 40013480 PMC11866988

[24] Wang J Hatt SR O'Connor AR Drover JR Adams R Birch EE . Final version of the distance randot stereotest: normative data, reliability, and validity. J AAPOS. (2010) 14:142–6. doi: 10.1016/j.jaapos.2009.12.159, 20199880 PMC2866770

[25] Seol BR Choung HK Kim SJ. Stereopsis before and after inferior oblique weakening surgery. Korean J Ophthalmol. (2018) 32:134–9. doi: 10.3341/kjo.2016.0105, 29560617 PMC5906398

[26] Niechwiej-Szwedo E Thai G Christian L. Contribution of stereopsis, vergence, and accommodative function to the performance of a precision grasping and placement task in typically developing children age 8-14 years. Hum Mov Sci. (2020) 72:102652. doi: 10.1016/j.humov.2020.102652, 32721372

[27] Hong EH Yang HK Kim JH Hwang JM. Bilateral fundus excyclotorsion in unilateral superior oblique palsy confirmed by MR imaging. J Clin Med. (2020) 9:1829. doi: 10.3390/jcm9061829, 32545329 PMC7356771

[28] Cha EH Ha SG Suh Shu Y Kim SH. Clinical features of excyclotorsion in the non-paretic eye of patients with congenital unilateral superior oblique palsy. BMC Ophthalmol. (2022) 22:126. doi: 10.1186/s12886-022-02339-5, 35296286 PMC8928676

[29] Kang H Lee SJ Shin HJ Lee AG. Measuring ocular torsion and its variations using different nonmydriatic fundus photographic methods. PLoS One. (2020) 15:e0244230. doi: 10.1371/journal.pone.0244230, 33351818 PMC7755211

[30] Lee LC Chang HC Chen YH Chien KH. A simple marking system for accurate intraoperative monitoring and adjustment of cyclotorsion strabismus surgery. Front Med (Lausanne). (2023) 9:1059790. doi: 10.3389/fmed.2022.1059790, 36687453 PMC9853205

[31] Lin HY Wu WC Sun MH Lin JY Huang PH Liu CH. Measurements of objective cyclotorsion in a population of healthy children. J Ophthalmol. (2024) 2024:6982201. doi: 10.1155/joph/6982201, 39734399 PMC11671662

[32] Liu R Zhao J Yu K Zhu D. The consistency and efficacy of optical coherence tomography for the evaluation of ocular torsion angle in children. Front Pediatr. (2025) 13:1519017. doi: 10.3389/fped.2025.1519017, 39950158 PMC11821915

[33] Shin KH Lee HJ Lim HT. Ocular torsion among patients with intermittent exotropia: relationships with disease severity factors. Am J Ophthalmol. (2013) 155:177–82. doi: 10.1016/j.ajo.2012.07.011, 23022165

