# Supplementary figures and images for: Stereopsis impairment and its association with fovea-disc angle in congenital superior oblique palsy patients with compensatory head posture: a cross-sectional study
Source: Front Med (Lausanne). 2026 Jun 12;13:1851621. doi: 10.3389/fmed.2026.1851621 (PMC13303132; doi:10.3389/fmed.2026.1851621)

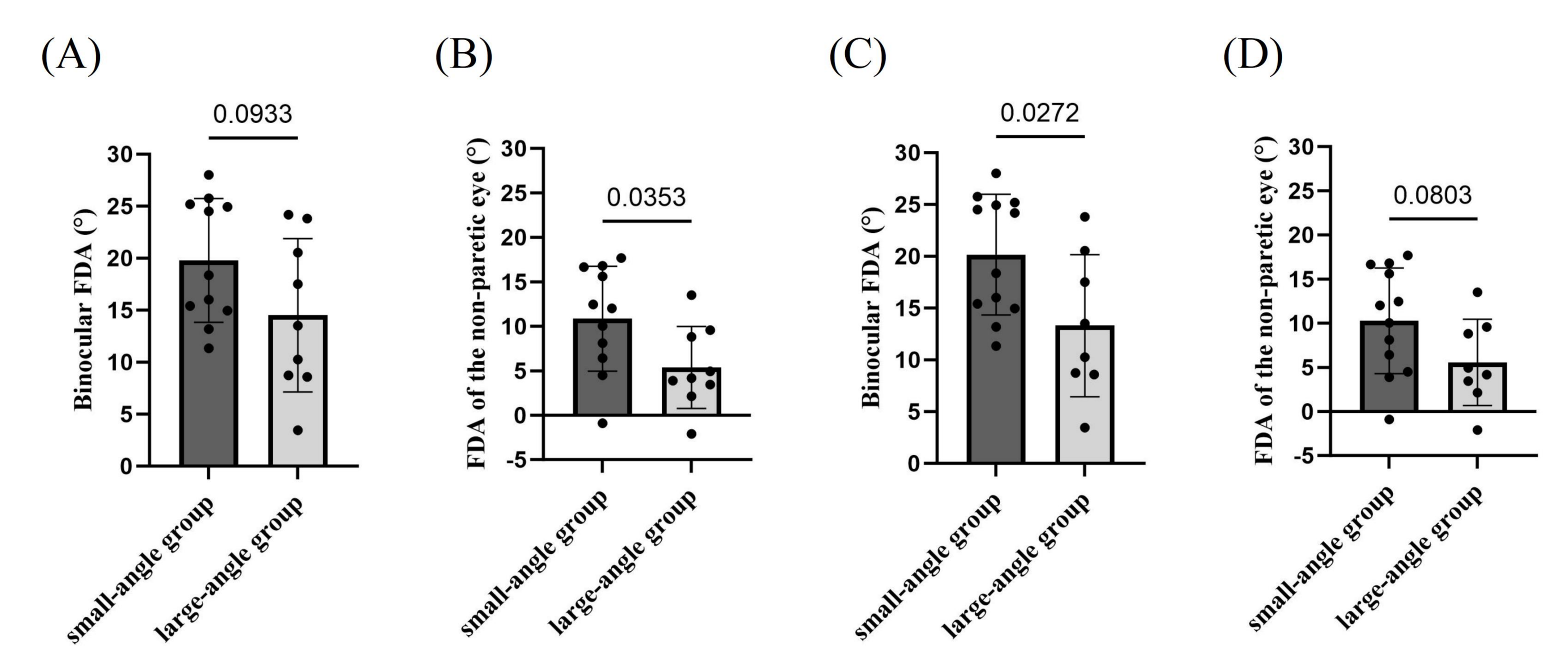

Supplement: Supplementary file 1 [file image_1.tif]

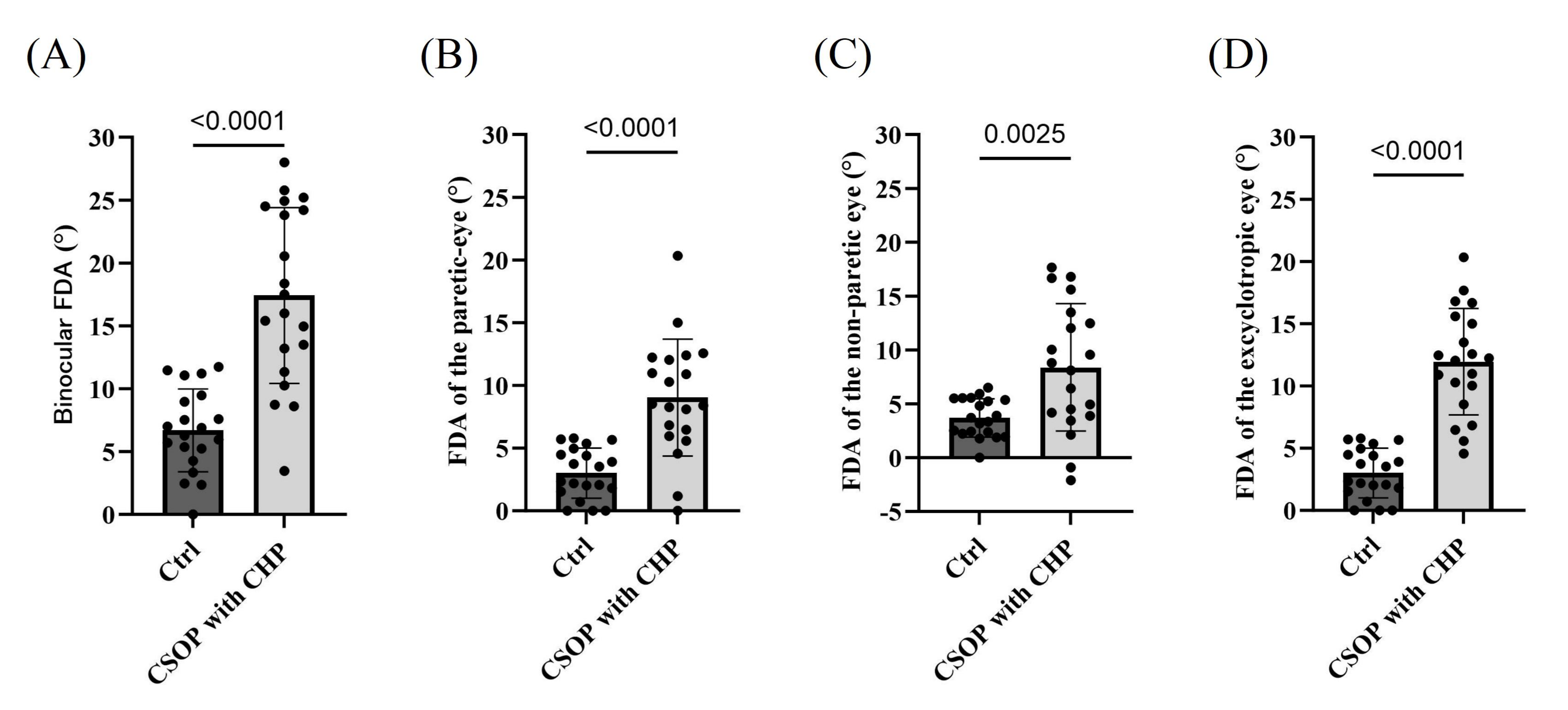

Supplement: Supplementary file 2 [file image_2.tif]
