# Supplementary material for: Stereopsis impairment and its association with fovea-disc angle in congenital superior oblique palsy patients with compensatory head posture: a cross-sectional study
Source: Front Med (Lausanne). 2026 Jun 12;13:1851621. doi: 10.3389/fmed.2026.1851621 (PMC13303132; doi:10.3389/fmed.2026.1851621)
Supplement: Supplementary file 3 [file supplementary_file_1.docx]

**Supplementary material:**

**Supplementary file 1**

**File format:** Excel.xlsx

**Title of data:** Table S1. Comparison of general data and stereopsis between the CSOP group and the control group.

**Description of data:**

Data are presented as *n* (%), mean ± standard deviation (SD), or median (interquartile range, IQR), as appropriate. Stereopsis in CSOP group was measured under compensatory head posture. Interocular differences are calculated as right-minus-left differences. Group comparisons were performed using the Fisher's exact test for categorical variables, independent samples *t* test for normally distributed continuous variables, and the Mann-Whitney *U* test for non-normally distributed continuous variables. For independent samples *t* test, the effect size and its 95% *CI* were reported; for the Mann-Whitney *U* test, the median difference and its approximate 95% *CI* were reported. CSOP, congenital superior oblique palsy; SE, spherical equivalent; D, diopters; SD, standard deviation; IQR, interquartile range; CI, confidence interval. Bold *p* indicates statistical significance (*p* < 0.05).

**Supplementary file 2**

**File format:** Excel.xlsx

**Title of data:** Table S2. Comparison of stereopsis under the CHP versus the primary position in CSOP patients.

**Description of data:**

Data are presented as median (interquartile range, IQR). All comparisons used Wilcoxon signed-rank test, the median difference and its approximate 95% *CI* were reported. CSOP, congenital superior oblique palsy; CHP, compensatory head posture; IQR, interquartile range; CI, confidence interval. Bold *p* indicates statistical significance (*p* < 0.05).

**Supplementary file 3**

**File format:** Excel.xlsx

**Title of data:** Table S3. Vertical deviation and FDA in CSOP patients with CHP.

**Description of data:**

Data are presented as mean ± standard deviation (SD). Paired sample *t* test was used for the comparison of FDA between eyes, the effect size and its 95% *CI* were reported; Wilcoxon signed-rank test was used for other comparisons, the median difference and its approximate 95% *CI* were reported. CSOP, congenital superior oblique palsy; CHP, compensatory head posture; FDA, fovea-disc angle; PD, prism diopters; SD, standard deviation; CI, confidence interval.

**Supplementary file 4**

**File format:** Excel.xlsx

**Title of data:** Table S4. Correlation analysis of stereopsis with clinical factors in CSOP patients with CHP.

**Description of data:**

Titmus and Randot distance were analyzed using Spearman correlation analysis with all clinical factors. Randot near was analyzed using Spearman correlation analysis with astigmatism of the right eye and the left eye, interocular difference in SE and astigmatism, and inferior oblique overaction; while all other analyses used Pearson correlation analysis. Stereopsis in CSOP group was measured under compensatory head posture. Higher log stereoacuity values indicate worse stereopsis; therefore, the observed negative correlation indicates that a smaller binocular FDA is associated with relatively worse stereopsis in our cohort. CSOP, congenital superior oblique palsy; CHP, compensatory head posture; FDA, fovea-disc angle; PD, prism diopters; SE, spherical equivalent; D, diopters; CI, confidence interval. Uncorrected *p* values are shown for exploratory purposes.
