# Supplementary material for: Stereopsis impairment and its association with fovea-disc angle in congenital superior oblique palsy patients with compensatory head posture: a cross-sectional study
Source: Front Med (Lausanne). 2026 Jun 12;13:1851621. doi: 10.3389/fmed.2026.1851621 (PMC13303132; doi:10.3389/fmed.2026.1851621)
Supplement: Supplementary file 4 [file supplementary_file_2.docx]

**Supplementary Figure S1**

**File format:** TIF


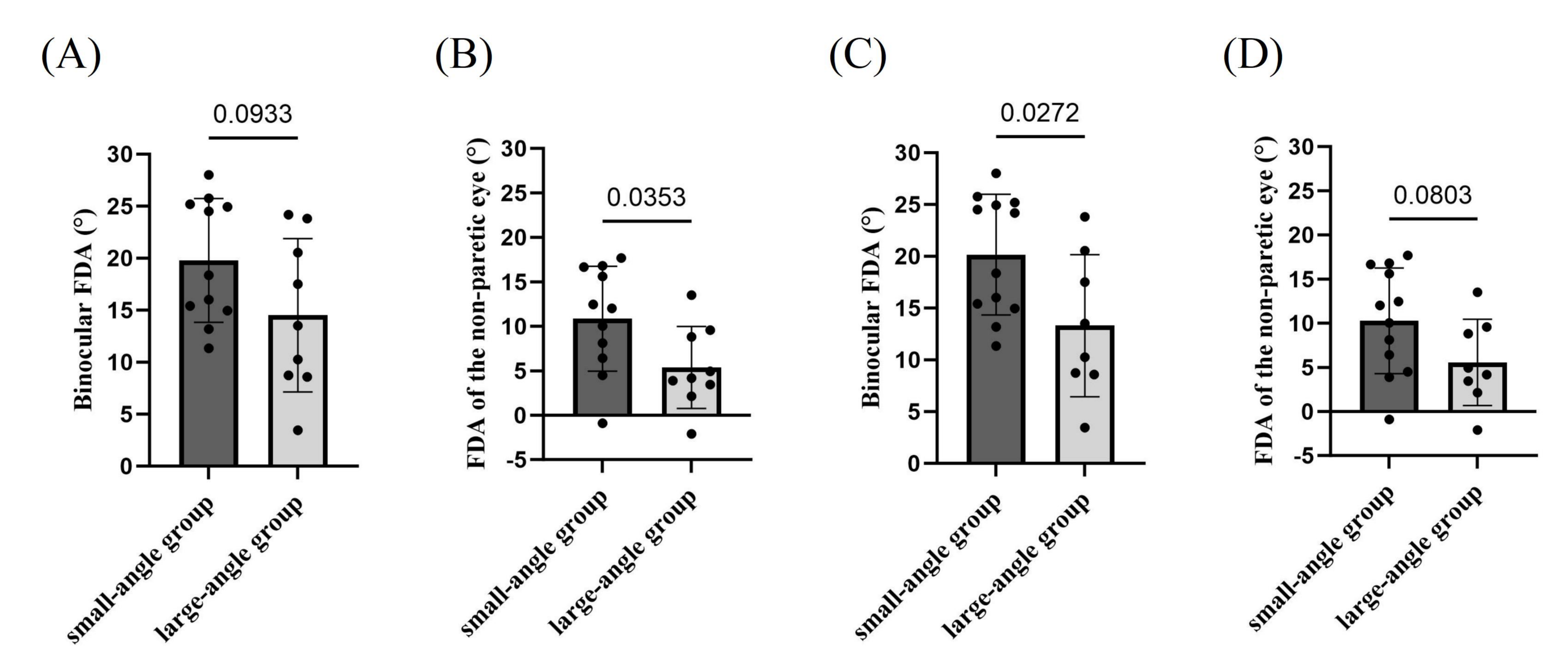


**Title of data:** Supplementary Figure S1. Comparisons of FDA parameters between the subgroups of small and large vertical deviation angles in CSOP patients with CHP.

**Description of data:**

(A-B) Comparisons of binocular FDA (A) and FDA of the non-paretic eye (B) between the subgroups of small and large distance vertical deviation angles; (C-D) Comparisons of binocular FDA (C) and FDA of the non-paretic eye (D) between the subgroups of small and large near vertical deviation angles. CSOP patients with CHP are stratified according to vertical deviation angles of the paretic eye in the primary position: the large-angle group (≥15 PD) and the small-angle group (<15 PD). These analyses are post-hoc and exploratory in nature. At distance: the large-angle group shows a significantly smaller FDA of the non-paretic eye than the small-angle group, and binocular FDA shows a non-significant decreasing trend. At near: the large-angle group shows a significantly smaller binocular FDA, and FDA of the non-paretic eye shows a non-significant decreasing trend. CSOP, congenital superior oblique palsy; CHP, compensatory head posture; FDA, fovea-disc angle; PD, prism diopters. Statistical significance is indicated by the *p* values above each comparison.

**Supplementary Figure S2**

**File format:** TIF


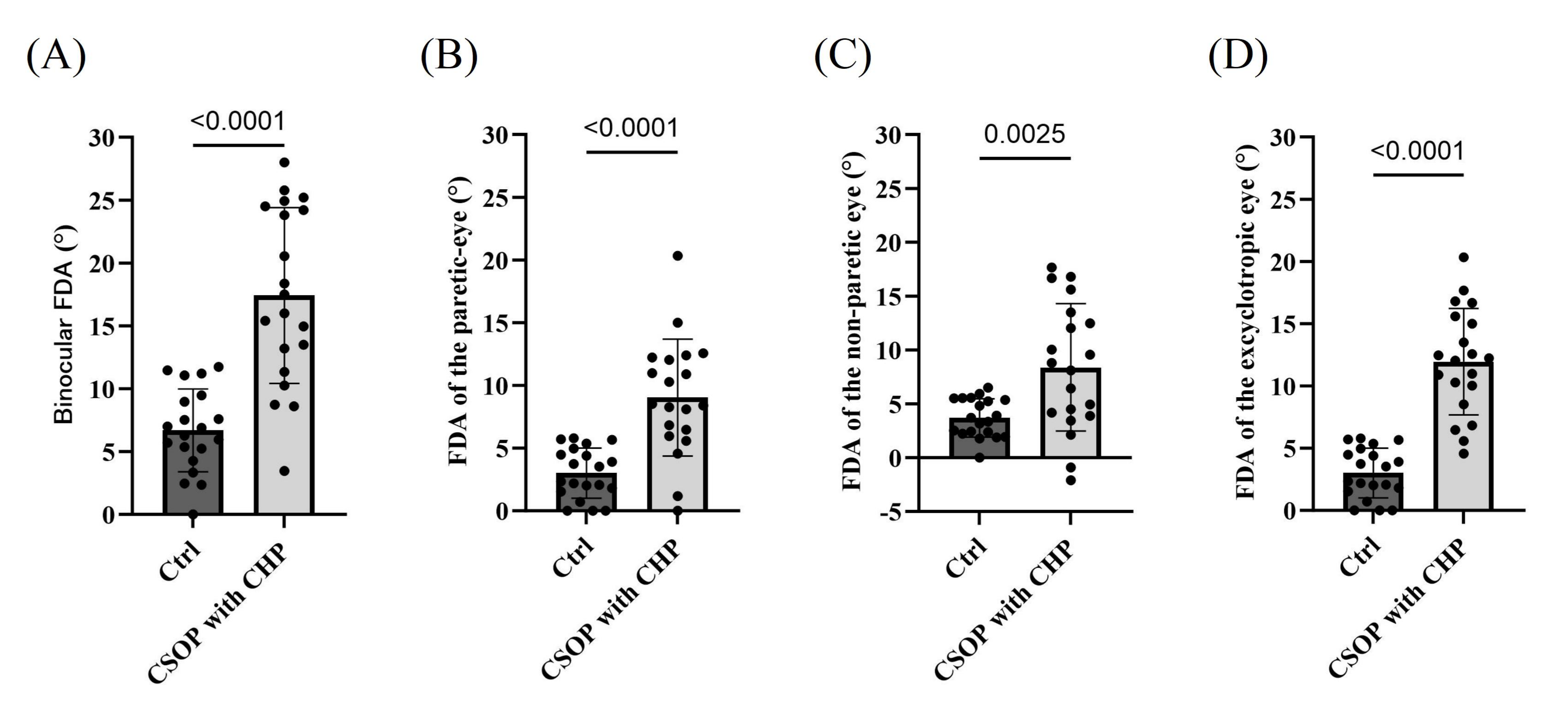


**Title of data:** Supplementary Figure S2. Comparisons of FDA parameters between CSOP patients with CHP and normal controls.

**Description of data:**

(A-D) Comparisons of binocular FDA (A), FDA of the paretic eye (B), FDA of the non-paretic eye (C), FDA of the excyclotropic eye (D) between the two groups. These analyses are post-hoc and exploratory in nature. In CSOP patients, the eye with greater excyclotorsion is defined as the excyclotropic eye. Normal controls showed no significant interocular FDA difference. For comparisons of monocular FDA, the right eye in the control group is used for comparison with the paretic eye and the excyclotropic eye of CSOP patients, and the left eye is used for comparison with the non-paretic eye of CSOP patients. The results indicate an overall excyclotorsion status in CSOP patients with CHP. CSOP, congenital superior oblique palsy; CHP, compensatory head posture; FDA, fovea-disc angle. Statistical significance is indicated by the *p* values above each comparison.
